# Impact of a Homestead Food Production program on poultry rearing and egg consumption: A cluster‐randomized controlled trial in Bangladesh

**DOI:** 10.1111/mcn.13505

**Published:** 2023-03-24

**Authors:** Nathalie J. Lambrecht, Jillian L. Waid, Amanda S. Wendt, Shafinaz Sobhan, Abdul Kader, Sabine Gabrysch

**Affiliations:** ^1^ Charité—Universitätsmedizin Berlin, Corporate Member of Freie Universität Berlin and Humboldt‐Universität zu Berlin, Institute of Public Health Berlin Germany; ^2^ Research Department 2 Potsdam Institute for Climate Impact Research (PIK), Member of the Leibniz Association Potsdam Germany; ^3^ Heidelberg Institute of Global Health Heidelberg University Heidelberg Germany; ^4^ Bangladesh Country Office, Helen Keller Intl Dhaka Bangladesh

**Keywords:** animal‐source foods, children, nutrition, nutrition‐sensitive agriculture, small‐scale poultry production, women

## Abstract

Women and children in Bangladesh face high levels of micronutrient deficiencies from inadequate diets. We evaluated the impact of a Homestead Food Production (HFP) intervention on poultry production, as a pathway outcome, and women's and children's egg consumption, as secondary outcomes, as part of the Food and Agricultural Approaches to Reducing Malnutrition cluster‐randomized trial in Sylhet division, Bangladesh. The 3‐year intervention (2015−2018) promoted home gardening, poultry rearing, and nutrition counseling. We randomly allocated 96 clusters to intervention (48 clusters; 1337 women) or control (48 clusters; 1368 women). Children < 3 years old born to participants were enrolled during the trial. We analyzed poultry production indicators, measured annually, and any egg consumption (24‐h recall), measured every 2−6 months for women and their children. We conducted intention‐to‐treat analyses using mixed‐effects logistic regression models with repeat measures, with minimal adjustment to increase precision. Poultry ownership increased by 16% points (pp) and egg production by 13 pp in the final intervention year. The intervention doubled women's odds of egg consumption in the final year (Odds Ratio [OR]: 2.31, 95% CI: 1.68−3.18), with positive effects sustained 1‐year post‐intervention (OR: 1.58, 95% CI: 1.16−2.15). Children's odds of egg consumption were increased in the final year (OR: 3.04, 95% CI: 1.87−4.95). Poultry ownership was associated with women's egg consumption, accounting for 12% of the total intervention effect, but not with children's egg consumption. Our findings demonstrate that an HFP program can have longer‐term positive effects on poultry production and women's and children's diets.

## Key messages

- The Food and Agricultural Approaches to Reducing Malnutrition trial evaluated a 3‐year Homestead Food Production intervention that promoted small‐scale poultry rearing, home gardening, and nutrition counseling among women's groups in rural Sylhet division, Bangladesh.
- The intervention increased poultry ownership, egg production, and improved poultry management practices.
- The intervention increased women's and children's egg consumption, with the strongest effects in year 3 of the intervention and evidence of sustained impacts post‐intervention.
- The impact of the Homestead Food Production program on women's egg consumption was partly mediated by increased poultry ownership.

## Graphical abstract

The Food and Agricultural Approaches to Reducing Malnutrition trial evaluated a 3‐year Homestead Food Production (HFP) intervention that promoted small‐scale poultry rearing, home gardening, and nutrition counseling among women's groups in rural Sylhet division, Bangladesh. The intervention increased poultry ownership and egg production, and women's and children's egg consumption. The impact of the HFP program on women′s egg consumption was partly mediated by increased poultry ownership.

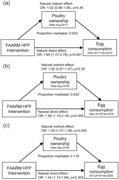

## 1 INTRODUCTION

Nearly 2.4 billion people globally do not have access to adequate diets (FAO IFAD UNICEF WFP and WHO, 2021). Young children and women of reproductive age in low‐income settings are especially vulnerable to micronutrient deficiencies, with serious adverse consequences for health and development (Black et al., 2013). South Asia has the largest number of children affected by stunting and wasting among all the world's regions (UNICEF WHO and World Bank Group, 2021). Children and women in this region face persistently high levels of zinc deficiency, anemia, and vitamin A deficiency (Harding et al., 2018). Modest increases in animal‐source food consumption may reduce micronutrient inadequacies among young children and women, whose higher nutrient requirements often cannot be met by their typical staple‐based diets (Beal et al., 2021; Lutter et al., 2018; Nordhagen et al., 2020; Ortenzi & Beal, 2021). However, animal‐source foods remain largely out of reach due to low availability and high cost (Morris et al., 2018; Ryckman et al., 2021).

Enhancing homestead production of livestock‐derived foods has the potential to improve diet quality and diversity (Ruel et al., 2018). While livestock transfer programs have traditionally focused on improving livelihoods, there has been growing interest in the potential of livestock rearing to improve maternal and child nutrition. For over three decades, the nonprofit Helen Keller International has promoted poultry production alongside home gardening as part of a Homestead Food Production (HFP) program to reduce undernutrition (Haselow et al., 2016). Several other recent nutrition‐sensitive interventions have promoted chicken production through training, the provision of indigenous or genetically improved chicks, and the provision of other inputs such as vaccination, chicken sheds, and market training (Gelli et al., 2018; Kumar et al., 2018; Leight et al., 2021; Marquis et al., 2018; McKune et al., 2020; Passarelli et al., 2020).

The interest in promoting poultry production in nutrition‐sensitive interventions is manifold. Foremost, household poultry can contribute to food availability directly through the production of meat and eggs and indirectly via the sale or exchange of poultry products for other foods (Alders et al., 2018; Iannotti et al., 2014; Wong et al., 2017). Chicken eggs contain essential fatty acids and protein, are rich in choline, vitamin A, vitamin B12, and zinc, and contribute to dietary diversity as a key food group (Iannotti et al., 2014). Second, in many low‐income countries, poultry are one of the few assets that women can own and control, enabling them to make decisions about the consumption or sale of chickens, meat, and eggs (Alders et al., 2018; Wong et al., 2017). Studies show that greater women's empowerment and control over resources have positive impacts on child nutrition (Heckert et al., 2019; Smith et al., 2003). Third, poultry are relatively affordable and accessible to resource‐constrained households as they require minimal land and inputs, are cheaper than other livestock to purchase, can scavenge for their own food, and propagate quickly (Alders et al., 2018; Iannotti et al., 2014; Wong et al., 2017). With modest investments in appropriate shelters, supplemental feed, and disease prevention and control, households can increase flock sizes and egg‐laying productivity (Alders et al., 2018; Wong et al., 2017).

Poultry production interventions have demonstrated positive impacts on children's diets, particularly when integrated with nutrition behavior change communication, according to several reviews (Chen et al., 2021; Ruel et al., 2018; Sharma et al., 2021). However, there is a lack of rigorous long‐term assessments of poultry interventions. To our knowledge, only two interventions have examined impacts on poultry production and diets beyond 2 years (Kadiyala et al., 2021; Rosenberg et al., 2018) and none have evaluated impacts post‐intervention. Given the volatility of poultry rearing due to disease, predation, and theft, but also the potential for poultry flocks to grow and provide greater benefits over time, more evidence is needed on the longer‐term potential of poultry rearing in agricultural interventions. In addition, there is limited research on the direct link between poultry rearing and egg consumption in poultry intervention studies.

In this article, we assess the impact of a multiyear HFP intervention on poultry production and women's and children's egg consumption during project implementation and 1‐year post‐intervention. We also evaluate the contribution of the poultry component of the HFP program to egg consumption at three time points: during, at the end, and after program activities.

## 2 METHODS

### 2.1 Study design and population

The Food and Agricultural Approaches to Reducing Malnutrition (FAARM) cluster‐randomized controlled trial was set up to evaluate the impact of an HFP program on women's and children's undernutrition in two sub‐districts of Habiganj district, Sylhet division, Bangladesh (ClinicalTrials.gov ID: NCT025‐05711). We used a cluster‐randomized design to facilitate the delivery of the intervention, using a woman farmer group approach, and to minimize the risk of contamination. The intervention promoted year‐round production and consumption of nutrient‐dense foods through home gardening, poultry rearing, and nutrition and hygiene education. Implementation was managed by the international nongovernmental organization Helen Keller International. The FAARM trial enrolled married women and their children 3 years of age or younger. Women were eligible if they reported to be 30 years of age or younger, had an interest in participating in the HFP program, and had access to at least 40 square meters of land. Each participant's youngest biological child was eligible for inclusion at baseline if they were born after March 2012, roughly 3 years before the baseline survey. During the intervention period, all children under 3 years of age born to participating women were eligible to be enrolled in the trial.

Settled areas in the Baniachong and Nabiganj subdistricts of Habiganj district with sufficient land and a minimum of 15 households were identified. Eligible women were then enumerated and 96 rural settlements (geographical clusters) were formed. Settlements had a minimum of 10 and no more than 65 eligible women and were based on the geographical location of women's residences. Settlements were separated by at least 400 meters to minimize the risk of spillover. The baseline survey was then conducted in which women were consented for participation and enrolled in the trial. After the baseline survey, covariate‐constrained randomization was used to allocate 48 settlements to receive the HFP intervention and 48 settlements to the control group, using the Stata command *ccrand* (Lorenz & Gabrysch, 2017) to ensure balance on important baseline characteristics, as described in the study protocol (Wendt et al., 2019). The study sample size was calculated based on the primary trial outcome, children's length/height‐for‐age z‐score, as described in the study protocol (Wendt et al., 2019). Outcome assessors were blinded to the intervention assignment and participants were not explicitly informed of their participation in the intervention group. Data analysts were not blinded to enable the project to track outcomes and adapt in light of data from the field, for example, the slow uptake of dietary change at the start of the program was investigated with qualitative interviews and monitoring of field facilitators, leading to hiring an additional field facilitator to reduce workload and adding a checklist to improve the breadth of counseling given. Further information about the FAARM trial design, intervention activities, and data collection can be found in the study protocol (Wendt et al., 2019).

### 2.2 Intervention

FAARM promoted small‐scale household poultry rearing alongside home gardening and nutrition counseling among the intervention households over 3 years, from mid‐2015 to mid‐2018, when field activities were phased out. One refresher training on nutrition was provided in mid‐2019. FAARM used a group leader approach, in which participants were organized into ‘women farmer groups’ and one lead farmer family oversaw a model farm and hosted trainings and meetings. Participating women received training on nutrition every 2 months, on vegetable production seasonally, and on poultry rearing annually. Full‐time project staff members led the training, and also conducted individual counseling sessions every 2 months to review key nutrition messages and provide technical support. Overall, training attendance averaged around 80%.

Poultry rearing: Training on poultry production included information on selecting healthy local poultry, building an ‘improved’ poultry shed with separate levels for the hen and chicks and adequate ventilation (Supporting Information: Figure 1), meeting nutrition needs of poultry with supplemental feed, as well as on poultry diseases, deworming, and vaccinations. FAARM promoted brooding and hatching practices to increase egg production, including the use of hatching pots (Hazals) (Supporting Information: Figure 2), optimal timing of chick separation from their mothers (within 7−10 days), and creep feeding (supplementing the diet of young chicks). Participants were encouraged to purchase 3−4 poultry from local breeders, received partial reimbursement for constructing an improved poultry shed, and received 2 kg of starter feed. FAARM also supported participants in vaccinating poultry against common diseases including Newcastle Disease, Fowl Cholera, Fowl Pox and Duck Plague through a community vaccination program. Project‐trained community vaccinators were equipped with vaccination supplies from the subdistrict livestock office and provided vaccinations to participating households via mass vaccination events and on an individual basis for a fee of 2−5 taka (approx. 0.05 USD) per bird.

Home gardening: Training was provided on year‐round vegetable cultivation and fruit tree production. Several gardening techniques were taught, including the use of raised beds, live fencing, pest management, composting and soil management, seed preservation, sack gardening, and production of urine‐enriched biochar fertilizer (Sutradhar, 2021). Seeds were provided to households twice yearly. Marketing training was provided in the third year of the intervention to support participants' income generation.

Nutrition counseling: Topics in nutrition, health, and hygiene were covered using an adult learning approach. Training followed the global Essential Nutrition Actions approach including sessions on breastfeeding, infant and young child feeding, maternal nutrition, and sick child care (World Health Organization, 2013). From June 2017 to February 2018, an additional food hygiene component was added which emphasized hand washing, washing feeding utensils, safe food and water storage, and cooking fresh or reheating food before feeding (Sobhan, 2022).

### 2.3 Data collection

Surveys were conducted by trained data collection officers using face‐to‐face interviews. All data were collected using tablet‐based Open Data Kit (ODK) software (Hartung et al., 2010). This study uses data from panel surveys conducted during the FAARM trial, including: (1) household listing conducted from July to December 2014 (enumeration), (2) baseline survey conducted from March to May 2015, (3) routine assessments conducted as part of a surveillance system every two months from September 2015 to September 2019, and (4) endline survey conducted from October 2019 to February 2020 (Supporting Information: Figure 3). The routine assessment which was conducted in 2019 and the endline survey captured participant data in the year after the completion of intervention activities.

Data on livestock ownership before the intervention and baseline household characteristics were extracted from the household listing and baseline surveys. Poultry production and management indicators were collected three times during surveillance rounds from May to August (2017 to 2019). Annually, each woman was asked about the number of adult chickens or ducks she owned, the number of eggs laid by her poultry in the past week, when she had last vaccinated her poultry, and whether she owned a poultry shed. Enumerators also conducted observations of whether the shed was improved or traditional and whether it was actually used for poultry.

Data on women's diets in the prior 24 h were collected every 6 months during the routine assessment period, with one‐third of women surveyed every round (i.e., 2 months). Data on children's diets were collected every 2 months up to 18 months of age and every 6 months thereafter, up to shortly after 3 years of age. At endline, diet data were collected from all women and their children under 24 months of age. Women's and children's diets were measured using a dietary diversity assessment. Briefly, women were asked to recall all the foods they had consumed in the previous day and night. Recalled food items were classified into 21 food groups by the enumerator who then inquired about groups not mentioned to verify that no food groups were missed (FAO and FHI 360, 2016). Children's diets were assessed according to the WHO Infant and Young Child Feeding survey module (World Health Organization, 2010). Mothers were asked to list all foods their child had consumed in the prior day and night, and foods were then categorized into food groups and verified.

### 2.4 Variables

Outcomes assessed in this study were poultry ownership, egg production, poultry vaccination and poultry shed use, which were prespecified pathway indicators of the trial. We then examined women's and children's egg consumption, which were pre‐specified secondary outcomes of the trial. All outcomes were assessed at the individual level. Poultry production was measured with two dichotomous and one continuous variable: ownership of any poultry (chickens or ducks), ownership of at least three poultry—the minimum target promoted by the FAARM intervention, and the number of poultry owned—capped at 95 chickens and ducks to reduce the influence of outlying larger‐scale poultry operations (<0.5% of all households in each of the three surveillance rounds). Egg production was assessed as the number of eggs produced per week, capped at 95 eggs, and as two dichotomous variables: production of at least one egg in the last week and at least seven eggs in the last week. Vaccination was assessed with two dichotomous variables: whether poultry were ever vaccinated and whether they were vaccinated in the prior 6 months. Poultry shed use was measured by three dichotomous variables: whether households owned a poultry shed, whether the shed was actively used for poultry, and whether they owned an ‘improved’ poultry shed. To evaluate egg consumption, dichotomous variables were created based on whether children consumed any egg and whether women consumed at least 15 g of egg (about one‐third of an egg) in the past 24 h.

Covariates included household religion, household wealth quintile at baseline, woman's education, child sex and age, month of survey and whether the day of recall occurred during Ramadan. A household wealth index was calculated using principle components analysis of household assets in line with standard demographic and health survey (DHS) techniques (Rutstein & Johnson, 2004) which was then categorized into wealth quintiles. For women enrolled in the second year for whom baseline data were not available, we used the wealth quintile of the household of which they became a member. For women with incomplete data at baseline, we imputed the wealth quintile from the average wealth quintile of the cluster, rounded to the nearest integer. Baseline wealth was also calculated in comparison to the 2014 Bangladesh DHS national wealth quintiles according to the Equity Tool guidelines (Metrics for Management, 2016). Children's age was calculated based on the date of birth recorded soon after birth or maternal recall otherwise. Women's education at baseline was categorized into those who had no formal education, partial primary education, complete primary education, or any secondary education based on the woman's reported number of school years completed.

### 2.5 Statistical analysis

We conducted descriptive analyses of household and women's characteristics at baseline and children's age and sex among the control and intervention groups using proportions for categorical variables and means and standard deviations for continuous variables. We evaluated the effects of the FAARM intervention on poultry ownership, egg production, vaccination practices, and poultry shed use using mixed‐effects regression models, accounting for clustering at the settlement level. We then estimated the effects of the intervention on women's and children's egg consumption in the past 24 h using mixed‐effects logistic regression models with random effects to account for settlement‐level clustering and repeated woman/mother and child measures. Egg consumption models included fixed effects for intervention group, as well as for month of survey and Ramadan, with child‐level models additionally adjusted for child sex, child age in days, and the square root of child age, selected a priori as strong predictors to increase precision of the estimates. All child models used probability weighting to account for less frequent surveillance of children older than 18 months. To estimate the effects of the intervention in 6‐month periods, we included an intervention group by time period interaction term. We then used the *lincom* command to calculate point estimates for each time period and exponentiated the coefficients to obtain odds ratios (OR) and 95% confidence intervals (CI). In a sensitivity analysis, we additionally adjusted for wealth quintile to account for the slight imbalance in baseline wealth between trial arms.

In an additional observational analysis, we examined the association between poultry ownership, defined as ownership of ≥3 poultry, and women's and children's egg consumption. We used mixed effects logistic regression models controlling for intervention group, religion, wealth quintile, women's education, and child sex and age, and random effects to account for clustering and repeat measures. Covariates were chosen a priori as factors which could influence poultry ownership and egg consumption based on existing literature. An interaction term was used to assess the effect of owning poultry on egg consumption in the 6 months following the routine surveys on poultry ownership (e.g., the association of poultry ownership at the end of year 2 [May−August 2017] with egg consumption in the subsequent 6 months [September 2017−February 2018]).

Causal mediation analysis was conducted using the ‘medflex’ package for R (Steen et al., 2017). We used the imputation‐based approach to calculate the natural direct and indirect effect of the HFP intervention on egg consumption mediated through women's ownership of ≥3 poultry. The indirect effect is interpreted as the change in the odds of egg consumption comparing poultry ownership observed without the intervention versus what it would have been with the intervention. The direct effect reflects the rest of the total effect of the HFP intervention on egg consumption, mediated through pathways other than poultry ownership. Models were adjusted for religion, household wealth quintile, and women's education to account for potential mediator‐outcome confounding. Standard errors were calculating using a bootstrapping method with 1000 iterations to account for clustering at the settlement level. Further information on the model specifications is provided in Supporting Information: Appendix 1.

Mediation analysis was conducted using R version 4.1.2 (R Core Team, 2021), and all other statistical analyses were conducted using Stata MP version 16.1 (StataCorp).

## 3 RESULTS

**Figure 1:**
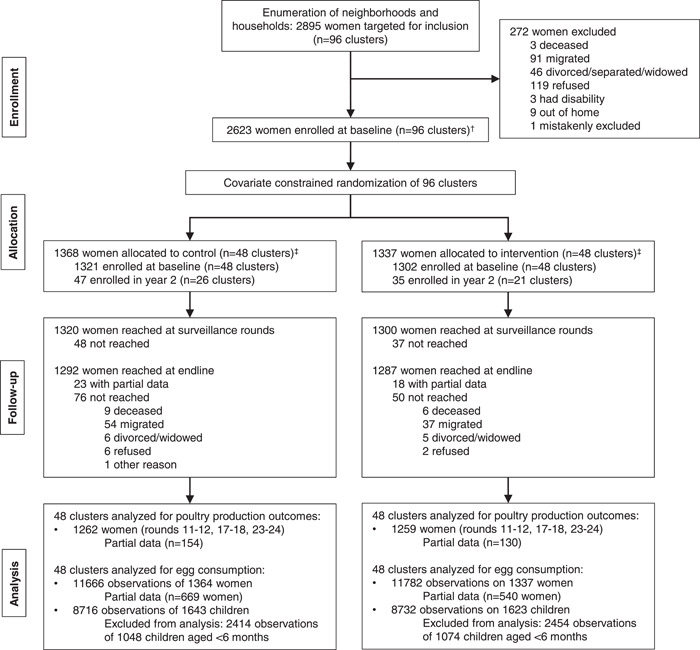
Trial profile and analytic sample selection. ^†^Women and their children were enrolled before randomization. For children born after the baseline survey, parental consent was taken at birth. ^‡^In year 2, women who were newly married into enrolled households were recruited for participation. In the control arm, 47 women in 26 settlements were enrolled out of 61 women in 30 settlements identified for recruitment. In the intervention arm, 35 women in 21 settlements were enrolled out of 53 women in 30 settlements identified for recruitment.

Overall, 2705 women were enrolled in the FAARM trial, with 1368 women allocated to the control group and 1337 to the intervention group (Figure 1). During the surveillance rounds, 2620 women were reached for data collection. At endline, 2579 (95%) were reached for data collection (control: 94%, intervention: 96%). At least one round of poultry surveillance data was available for 2521 women. We analyzed egg consumption data from 23,448 observations of 2701 women and 17,448 observations of 3266 children in 96 clusters, excluding observations of children when they were less than 6 months old. The number of observations of women and children per survey round is included in Supporting Information: Table 1.

The intervention and control groups were similar concerning women's age and education level, and household religion, wealth, and livestock ownership at baseline (Table 1). Approximately one‐third of households were Hindu and two‐thirds were Muslim. Women were on average 25 years old and over half of women had completed at least primary education. Relative to the 2014 Bangladesh national wealth index, approximately half of households belonged to the low and middle quintiles and one‐quarter to the high wealth quintile. A slightly higher proportion of households in the intervention group belonged to the upper wealth quintiles. At enumeration, approximately half of households owned cows, oxen or bulls, less than one‐fifth owned goats or sheep, over half owned chickens, and over one‐third owned ducks. Children were on average 21 months old across all survey rounds and slightly over half were male.

**Table 1:** Characteristics of households, women and their children at baseline.

Characteristic	Control	*n*	Intervention	*n*
*Household characteristics*				
Religion		1364		1337
Muslim	66.4		70.8	
Hindu	33.7		29.2	
National wealth quintile^a^		1345		1324
Lowest	15.5		11.0	
Low	31.9		26.0	
Middle	20.5		24.8	
High	26.3		31.7	
Highest	5.9		6.7	
Owns cows, oxen, or bulls^b^	54.0	1155	50.9	1131
Owns goats or sheep^b^	12.2	1164	16.9	1144
Owns chickens^b^	56.8	1164	55.4	1144
Owns ducks^b^	37.2	1164	35.9	1144
*Women's characteristics*				
Age, years	24.4 ± 4.4	1364	24.6 ± 4.3	1337
Education level		1364		1337
None	15.7		15.3	
Partial primary	22.4		21.8	
Complete primary	24.0		21.6	
Any secondary education	38.0		41.3	
Consumed eggs in past 24 h	13.7	1309	12.5	1290
*Child characteristics*				
Sex^c^		1643		1623
Male	51.3		50.5	
Female	48.7		49.5	
Age, months^d^	21.2 ± 8.9	8716	21.2 ± 8.9	8732
Consumed eggs in past 24 h	13.6	610	16.2	593

*Note*: Values are % for categorical variables or means ± SDs for continuous variables.

^a^ Wealth quintiles reflect the study population's wealth relative the national wealth quintiles in Bangladesh in 2014 using www.equitytool.org.

^b^ Livestock ownership data were collected in the enumeration survey (July to December 2014).

^c^ Includes children at baseline and those enrolled during the trial (2015−2019).

^d^ Child age is calculated as the average age of all included children over all survey rounds weighted to account for less frequent sampling of children above 18 months during surveillance rounds. In the control group this includes 8716 observations of 1643 children and in the intervention group this includes 8732 observations of 1623 children.

### 3.1 Impact of the HFP intervention on poultry production

During and after HFP activities, a higher proportion of women in the intervention group owned poultry and had poultry that produced eggs compared to the control group (Table 2). Poultry uptake among the intervention group was highest in the third year of the intervention, with 74% of intervention women owning any poultry and 48% owning at least three chickens or ducks, versus 58% and 29%, respectively, among the control group (*p* < 0.001). Post‐intervention, poultry ownership declined in the intervention group but remained increased compared to controls. On average, women with poultry in the intervention group had 0.6 (95% CI: 0.2−1.1) more chickens in the final year and 0.4 (95% CI: 0.0−0.8) more chickens post‐intervention. During and after the intervention, approximately one‐third of women in the intervention group reported that their poultry produced at least one egg in the past week, compared to one‐fifth of control women (*p* < 0.001) (Table 2). Over all three surveillance rounds, women's poultry in the intervention group produced on average an additional one to two eggs per week.

**Table 2:** Impact of a Homestead Food Production intervention on poultry production and management indicators.

	Year 2, May−August 2017, *n* = 2453				Year 3, May−August 2018, *n* = 2380				Scale‐down, May−September 2019, *n* = 2347			
	Con.	Int.	Effect estimate (95% CI)	*p* Value	Con.	Int.	Effect estimate (95% CI)	*p* Value	Con.	Int.	Effect estimate (95% CI)	*p* Value
Poultry ownership												
Poultry ownership, %	49.6	69.1	2.39 (1.58−3.61)	<0.001	58.4	74.2	2.24 (1.53−3.30)	<0.001	55.2	65.4	1.53 (1.10−2.12)	0.012
Owns ≥ 3 poultry, %	26.6	40.3	1.95 (1.45−2.63)	<0.001	28.8	47.5	2.38 (1.79−3.17)	<0.001	25.0	35.9	1.71 (1.29−2.26)	<0.001
Owns chickens, %	41.6	60.5	2.56 (1.45−4.51)	0.001	49.2	63.8	2.23 (1.23−4.03)	0.008	42.8	54.9	1.79 (1.07−3.00)	0.027
Avg. number of chickens (among poultry owners)^a^	2.3	2.5	0.31 (−0.13 to 0.76)	0.17	2.3	2.8	0.62 (0.17−1.07)	0.007	1.9	2.3	0.42 (0.04−0.79)	0.029
Owns ducks, %	20.1	27.1	1.45 (1.09−1.94)	0.011	21.5	29.2	1.54 (1.19−2.01)	0.001	19.1	25.0	1.43 (1.10−1.87)	0.007
Avg. number of ducks (among poultry owners)^a^	2.8	2.2	−0.58 (−1.79 to 0.63)	0.35	2.0	2.3	0.33 (−0.55 to 1.20)	0.46	1.7	2.1	0.35 (−0.22 to 0.91)	0.227
Egg production												
Household produced any eggs in past week, %	19.9	30.0	1.73 (1.27−2.37)	0.001	22.4	35.5	1.91 (1.50−2.44)	<0.001	21.7	31.8	1.73 (1.34−2.23)	<0.001
Household produced ≥ 7 eggs in past week, %	15.3	20.9	1.48 (1.06−2.05)	0.02	17.8	27.9	1.82 (1.39−2.38)	<0.001	16.7	23.9	1.63 (1.20−2.20)	0.002
Avg. number of eggs produced in past week	2.9	4.1	1.12 (0.02−2.23)	0.047	3.0	5.1	2.13 (0.90−3.35)	0.001	2.9	4.3	1.39 (0.37−2.40)	0.007
Number of eggs per poultry produced in last week (among poultry owners)^b^	1.2	1.2	0.01 (−0.24 to 0.25)	0.97	1.2	1.4	0.14 (−0.16 to 0.45)	0.35	1.2	1.5	0.34 (0.05−0.63)	0.02
Vaccination practices												
Poultry never vaccinated (among poultry owners)^a^, %	97.4	58.5	0.02 (0.01−0.06)	<0.001	98.0	47.6	0.01 (0.01−0.03)	<0.001	93.0	42.1	0.02 (0.01−0.05)	<0.001
Poultry vaccinated in past 6 months (among poultry owners)^a^, %	2.1	34.8	35.89 (13.26−97.17)	<0.001	0.9	26.9	53.40 (19.90−143.3)	<0.001	5.5	14.6	4.16 (1.91−9.06)	<0.001
Poultry shed use												
Owns poultry shed, %	5.9	52.5	25.91 (15.47−43.39)	<0.001	27.9	76.9	13.93 (7.98−24.33)	<0.001	9.3	60.8	27.25 (15.11−49.15)	<0.001
Owns ‘improved’ poultry shed^c^, %	0.2	45.3	–	–	0.2	67.8	–	–	0.4	55.3	–	–
Poultry shed is used for poultry (among poultry owners)^d^, %	10.5	50.4	11.02 (6.86−17.70)	<0.001	46.8	74.9	4.00 (2.10−7.60)	<0.001	15.3	53.2	9.45 (5.63−15.86)	<0.001

*Note*: Values for control (con.) and intervention (int.) groups are % or mean. Effect estimates are odds ratios (95% CI) or mean differences (95% CI) using mixed effects regression models. Clustering is accounted for using random effects at the settlement level. Analyses are intention‐to‐treat. ‘–’ Indicates model not evaluated because of <1% prevalence in one of the groups.

^a^ Year 2: *n* = 1456; Year 3: *n* = 1579; Scale‐down: *n* = 1417.

^b^ Year 2: *n* = 1439; Year 3: *n* = 1539; Scale‐down: *n* = 1333.

^c^ An improved poultry shed was defined as well‐ventilated, with more than one room, easy to clean, and well‐secured. Year 2: *n* = 2452; Year 3: *n* = 2370; Scale‐down: *n* = 2341.

^d^ Year 2: *n* = 1452; Year 3: *n* = 1575; Scale‐down: *n* = 1411.

The intervention also increased women's use of improved poultry management practices. The proportion of women in the intervention group who had vaccinated their poultry in the prior 6 months was highest in year 2 of the intervention (35%) and then decreased in the final year (27%) and post‐intervention (15%) (Table 2). In contrast, most women (93%−98%) in the control group had never vaccinated their poultry. Seventy‐seven percent of women in the intervention group owned a poultry shed in the final year compared to 28% of women in the control group (*p* < 0.001). Interestingly, there was fluctuation in shed ownership in the control group (6%−28%), though the reason for this is unclear. In the final year, 68% of all women in the intervention group owned an ‘improved’ shed and, among poultry owners, 75% used the shed for poultry. Post‐intervention, 75% of sheds in the intervention group had separate rooms for adult chickens and chicks, were well‐ventilated, and easy to clean, while 34% were off the ground, 27% had a water feeder, and 20% had a Hazal (hatching pot) (Supporting Information: Table 2).

### 3.2 Impact of the HFP intervention on egg consumption

At baseline, 13% of intervention and 14% of control women had consumed eggs in the 24 h before the survey (Table 1). The HFP intervention significantly increased women's egg consumption after 1 year with increases sustained post‐intervention (Table 3). The effect was highest in year 3, with intervention women having more than double the odds of consuming eggs (*p* < 0.001). The proportion of women who consumed eggs peaked at 25% in the intervention group in year 3, compared to 13% in the control group. We found that egg consumption remained increased after the completion of the intervention, though with slightly smaller effect sizes. One year post‐intervention, 20% of women in the intervention group compared to 14% in the control group had consumed eggs in the prior 24 h (OR: 1.58, 95% CI: 1.16−2.15). The results were stable to adjustment for baseline household wealth (Supporting Information: Table 3).

**Table 3:** Effect of a Homestead Food Production intervention on egg consumption among women and their children aged 6−36 months.

Time period^a^	Women					Children^b^				
	Con.	Int.	OR (95% CI)		*p* Value	Con.	Int.	OR (95% CI)		*p* Value
Intervention Y1, Sep 15−Feb 16	9.7%	12.1%	1.27 (0.90−1.80)		0.17	12.3%	15.9%	1.49 (0.91−2.44)		0.12
Intervention Y1, Mar 16−Aug 16	8.6%	10.2%	1.20 (0.83−1.73)		0.33	12.6%	15.2%	1.51 (0.84−2.72)		0.17
Intervention Y2, Sep 16−Feb 17	5.9%	10.3%	1.87 (1.27−2.75)		0.002	16.3%	15.4%	1.07 (0.55−2.09)		0.84
Intervention Y2, Mar 17−Aug 17	10.6%	16.3%	1.68 (1.20−2.36)		0.003	15.8%	21.4%	2.02 (1.34−3.04)		0.001
Intervention Y3, Sep 17−Feb 18	9.3%	17.4%	2.16 (1.54−3.03)		<0.001	12.4%	21.9%	3.04 (1.87−4.95)		<0.001
Intervention Y3, Mar 18−Aug 18	13.1%	24.5%	2.31 (1.68−3.18)		<0.001	14.9%	27.0%	2.90 (1.87−4.50)		<0.001
Scale‐down, Sep 18−Feb 19	10.7%	17.5%	1.82 (1.30−2.54)		<0.001	14.2%	22.7%	2.35 (1.41−3.92)		0.001
Scale‐down, Mar 19−Sep 19	11.3%	17.9%	1.75 (1.26−2.45)		0.001	14.2%	18.7%	1.89 (1.10−3.24)		0.02
Post‐intervention^c^, Oct 19−Feb 20	13.9%	19.8%	1.58 (1.16−2.15)		0.004	18.7%	24.4%	1.83 (0.94−3.55)		0.08
Total observations in model	20849					16245				
Total n in model	2670					2992				
ICC (cluster)	0.063					0.040				
ICC (woman)	0.196					0.150				
ICC (child)						0.494				

*Note*: Values are OR (odds ratio) and 95% CI (confidence interval) calculated using mixed‐effects logistic regression models with fixed effects for intervention group, month of survey, and Ramadan. An interaction term was used to calculate effects by half‐year. Clustering is accounted for using a random effect at the settlement level. Models additionally include random effects for woman/mother and child, if applicable, to account for repeated measures. Analyses are intention‐to‐treat.

Abbreviations: Con., Control; ICC, Intracluster correlation coefficient; Int., Intervention.

^a^ The first year of the intervention was from Sep. 2015 to Aug. 2016, the second year from Sep. 2016 to Aug. 2017, and third year from Sep. 2017 to Aug. 2018. The scale‐down of the intervention was from Sep. 2018 to Sep. 2019 during which field activities were phased out and stopped by Dec. 2018 and only a nutrition counseling refresher training was provided in May 2019. The post‐intervention endline survey was conducted from Oct. 2019 to Feb. 2020.

^b^ The model for children additionally includes covariates for age, square root of age, and sex, and probability weights to account for less frequent sampling of children > 18 months old during surveillance rounds.

^c^ The post‐intervention survey includes only children 6−24 months old.

Among children at baseline, 16% in the intervention and 14% in the control group consumed eggs in the past 24 h. The HFP intervention increased egg consumption significantly after 1.5 years, with strongest effects in the final year when children's odds of consuming eggs were tripled (Table 3). Egg consumption was highest among intervention children in the second half of year 3, when 27% had consumed eggs, compared to 15% in the control group. There was also evidence of sustained effects during the scale‐down period and post‐intervention. During scale‐down of project activities, the proportion of children consuming eggs remained higher in the intervention group. We found marginal evidence that egg consumption was still increased at endline, 1‐year post‐intervention (OR: 1.83, 95% CI: 0.94−3.55). Given the smaller sample size of the endline survey, we also examined the overall post‐intervention effect and found that egg consumption was increased during the scale‐down and post‐intervention periods combined (OR: 2.05, 95% CI: 1.38−3.06; *p* < 0.001) (Supporting Information: Table 4).

### 3.3 The effect of poultry ownership on egg consumption

**Figure 2:**
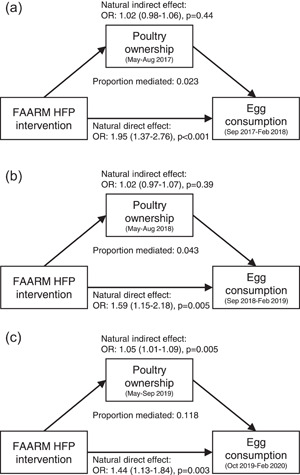
Mediation results of women's egg consumption through ownership of at least 3 poultry in the prior 6 months in (a) intervention year 3, (b) intervention scale‐down, and (c) post‐intervention. Causal mediation analysis was conducted with the ‘medflex’ package for R using the imputation‐based approach (Steen et al., 2017). Models were adjusted for religion, household wealth quintile, and women's education to account for potential mediator‐outcome confounding. Standard errors were calculating using a bootstrapping method with 1000 iterations to account for clustering at the settlement level.

Women's ownership of at least 3 poultry was marginally associated with egg consumption in year 3 and during scale‐down of the intervention in unadjusted models, but no association was observed after adjusting for intervention group, household religion and wealth, and women's education (Supporting Information: Table 5). However, we observed a positive association between women's poultry ownership and egg consumption in the post‐intervention period (adjusted OR: 1.58, 95% CI: 1.22−2.04). Using mediation analysis, we found evidence that poultry ownership at 6‐months post‐intervention significantly mediated the association between the HFP intervention and women's egg consumption at endline (one year post‐intervention), but not at other time points (Figure 2). The HFP intervention had a positive direct effect on women's egg consumption (OR: 1.44, 95% CI: 1.13−1.84) and positive indirect effect through poultry ownership (OR: 1.05, 95% CI: 1.01−1.09) (Figure 2c). Overall, women's ownership of at least 3 poultry explained 12% of the total effect of the HFP intervention on women's egg consumption at endline. In contrast, women's poultry ownership was not associated with their children's egg consumption (Supporting Information: Table 5). We therefore did not conduct a mediation analysis of the effect of the HFP intervention on children's egg consumption through poultry.

## 4 DISCUSSION

Our study evaluated an HFP intervention that promoted poultry rearing, home gardening, and nutrition counseling in women's groups over a 3‐year period, examining impacts during project implementation and 1 year after the conclusion of program activities. We found that the intervention improved poultry production and management practices and increased women's and children's egg consumption. We also demonstrated a sustained increase in egg consumption 1‐year post‐intervention, the first such evaluation to our knowledge to demonstrate longer‐term impacts. In women, this was in part attributable to increased poultry ownership. We did not find evidence that poultry mediated the impact of the intervention on children's egg consumption. This study presents the first results examining a program impact pathway of the FAARM trial. A comprehensive analysis examining multiple program impact pathways will be published separately.

As in our study, other poultry interventions have demonstrated increases in chicken and egg production (Alderman et al., 2022; Gelli et al., 2018; McKune et al., 2020; Passarelli et al., 2020), while some found no effects (Kadiyala et al., 2021; Leight et al., 2021; Nordhagen & Klemm, 2018; Olney et al., 2009; Rosenberg et al., 2018). We also found improvements in poultry vaccination practices and poultry shed use, similar to improvements in poultry management practices noted in other interventions (Leight et al., 2021; Passarelli et al., 2020). While our study provided support for building a poultry shed, vaccinating poultry, and training to improve production, we did not provide households with chickens or ducks. Most other poultry interventions provided poultry to participating households, ranging from a few to 25 vaccinated and improved‐breed chicks. Nevertheless, we found that the intervention increased poultry ownership by ~0.5 chickens and egg production by ~2 eggs per week, which is notable compared to increases of approximately 1−2 chickens and 3 eggs found by interventions in Malawi (Gelli et al., 2018) and Ethiopia (Passarelli et al., 2020) that gifted 10 and 25 chicks, respectively. To our knowledge, only two other studies reported on poultry impacts in a 3‐ (Kadiyala et al., 2021) and 4‐year intervention (Rosenberg et al., 2018), while the majority of studies to date assessed impacts after 1−2 years of intervention. Our study adds new evidence to the literature on poultry impact trials, demonstrating positive impacts on poultry production over a 3‐year trial with a sustained impact in the year after the conclusion of intervention activities.

In our study, egg consumption increased among women 1 year after the start of the intervention and remained higher than controls throughout and up to 1‐year post‐intervention. Two poultry intervention studies have also demonstrated positive impacts on women's egg consumption (Alderman et al., 2022; Nordhagen & Klemm, 2018) while others have found no effects on women's egg consumption (Olney et al., 2009) or their dietary diversity (Kadiyala et al., 2021; Rosenberg et al., 2018). The evidence linking poultry production to egg consumption in our study was mixed. While ownership of at least 3 poultry was not associated with egg consumption during the intervention, our results suggest that poultry ownership positively impacted women's egg consumption after intervention activities had concluded. Intervention households continued to produce more eggs compared to controls post‐intervention, which suggests that the poultry component of the HFP intervention was sustained and may have enabled some households to produce eggs for consumption. Nevertheless, we found that poultry production mediated only 12% of the intervention's impact on women's egg consumption. Indeed, egg production was low overall, with two‐thirds of intervention households reporting no egg production post‐intervention. Other HFP intervention components, including nutrition education and women's empowerment, may thus have contributed more to increasing egg consumption among women.

We found that the HFP intervention increased children's egg consumption 1.5 years into the intervention, with evidence that this positive impact was sustained post‐intervention. Several poultry trials have similarly reported positive impacts on children's egg consumption (Alderman et al., 2022; Becquey et al., 2022; McKune et al., 2020; Nordhagen & Klemm, 2018; Olney et al., 2009; Passarelli et al., 2020) while four studies found no effect (Boedecker et al., 2019; Kim et al., 2019; Marquis et al., 2018; Rosenberg et al., 2018). To our knowledge, our study is the first to report on children's egg consumption after the conclusion of a multiyear intervention. However, we did not find evidence that the increase in children's egg consumption was mediated through increased poultry production. This suggests that other program components, including nutrition counseling, were likely key to achieving higher egg consumption in our setting. Nutrition education is important to overcome taboos against children's egg consumption, misconceptions about the appropriateness of eggs for young children, and unequal intra‐household food allocation (Iannotti et al., 2014). Evidence from other studies suggests that nutrition education can act synergistically with poultry rearing to improve children's diets, providing a greater impact than either component alone (McKune et al., 2020; Passarelli et al., 2020).

The small contribution of the poultry component on egg consumption among women and children in our study may be due to some challenges faced during project implementation. Participants were slow to construct poultry sheds, delaying their uptake by about 1.5 years. Some participants were also reluctant to use the sheds, preferring to allow poultry to free‐roam outside during the day and keep them inside the house at night to prevent theft and predation, a barrier also noted in other Helen Keller International poultry interventions (Nordhagen & Klemm, 2018). There was also low retention of equipment, including water bottles and Hazals, post‐intervention. Our intervention also faced challenges in facilitating community vaccination, including limited mobility and benefit for women vaccinators and hesitation as to the benefits of vaccinations. Low participation in vaccination may have contributed to flock mortality and low overall productivity. Other poultry interventions have similarly reported challenges with sustaining community vaccination programs and reported high poultry mortality (Nordhagen & Klemm, 2018; Passarelli et al., 2022). In our study, Hindu households also feared that chickens would contaminate their indoor prayer areas, so some did not participate in chicken rearing and instead reared ducks or did not take part in this component of the intervention at all. While the FAARM trial also supported duck rearing, most activities were focused on chicken production. To achieve higher flock productivity, further work is needed to understand how to facilitate participation in improved production activities. Future poultry interventions should consider qualitative evaluations to better understand barriers and opportunities to poultry rearing, such as the evaluation conducted by Passarelli et al. (2022), and use participatory approaches to design interventions that are best suited for program participants.

Strengths of our study include the robust study design, comprehensive data collection during and post‐intervention, and a large sample size. Our study also had some limitations. We used 24‐h dietary recalls which may underestimate the intake of infrequently consumed foods such as eggs. Poultry and dietary data were based on women's self‐report, which may be subject to recall bias and social desirability bias as it was not possible to blind participants to the intervention. To understand the production‐consumption pathway, additional quantitative and qualitative data are needed on the use of poultry and poultry eggs for food, income, or other purposes, and the source of eggs for consumption, which was not analyzed in this study. Further, poultry production may have increased egg consumption in ways that we did not capture by only examining one indicator of poultry production in the mediation analysis. Additionally, our results may not be generalizable to other settings in Bangladesh, as women in our study area had generally limited mobility and rights, thus we may have seen a different impact if the trial had taken place in an area with higher women's empowerment. Finally, poultry production may increase the risk of zoonotic disease exposure in women and children (Alders et al., 2018), which we did not address in this study but plan to for future analyses.

## 5 CONCLUSION

To our knowledge, our study is the first to show positive impacts of a multiyear HFP program on poultry production and egg consumption both during and post‐intervention. While improved poultry production contributed to increased egg consumption among women, our results suggest that impacts were achieved primarily through other program components. Barriers related to shed use, vaccination, and other improved practices may have limited the potential of poultry production to contribute to diets. Further research on improving the design, delivery, and uptake of poultry interventions, with emphasis on participatory approaches that identify barriers and opportunities as well as provide assets that households most need, may enhance the potential of poultry to improve dietary outcomes. Further, our results highlight the need to evaluate the potential benefits and drawbacks of individual program components in complex, multicomponent interventions.

## AUTHOR CONTRIBUTION

Nathalie J. Lambrecht, Sabine Gabrysch, and Jillian L. Waid conceived and designed the analysis. Sabine Gabrysch, Jillian L. Waid, and Amanda S. Wendt contributed to the design and development of the FAARM trial. Sabine Gabrysch is the principal investigator of the FAARM trial. Abdul Kader oversaw data collection activities. Jillian L. Waid processed the data. Nathalie J. Lambrecht analyzed the data and wrote the first draft of the manuscript. Shafinaz Sobhan provided support in interpreting results. All authors contributed to the revision of the manuscript, and read and approved the final version.

## CONFLICT OF INTEREST STATEMENT

The authors declare no conflict of interest.

## ETHICS STATEMENT

The FAARM trial protocol was positively reviewed by the ethics committees of Heidelberg University in Germany and the James P Grant School of Public Health at BRAC University in Bangladesh. The study was conducted in accordance with the 1964 Helsinki Declaration and its later amendments or comparable ethical standards. All FAARM participants provided informed written consent by signature or thumbprint for themselves and their children (Wendt et al., 2019).

## Supporting information

Supporting information.

## Data Availability

A deidentified data set with the individual participant data that underlie the results reported in this article is available to interested researchers who provide a methodologically sound proposal for use of the data. Data requests with a proposal should be directed to the corresponding author (N. J. L.) and the principal investigator (S. G.; sabine.gabrysch@charite.de). A data access agreement will need to be signed to gain access to the data. The FAARM trial protocol is available online.
