# Supplementary material for: Impact of a Homestead Food Production program on poultry rearing and egg consumption: A cluster‐randomized controlled trial in Bangladesh
Source: Matern Child Nutr. 2023 Mar 24;19(3):e13505. doi: 10.1111/mcn.13505 (PMC10262892; doi:10.1111/mcn.13505)
Supplement: Supplementary file 1 — Supporting information. [file MCN-19-e13505-s001.docx]

**Supplemental Appendix**

# Appendix 1. Mediation model specification

We conducted causal mediation analysis using the imputation-based approach in the ‘medflex’ package (version 0.6-7) for R, as described in Steen et al. (2017). The outcome model was specified as a binomial logistic regression model, regressing the exposure (allocation to the Homestead Food Production intervention), mediator (ownership of at least 3 poultry), and mediator-outcome confounders (religion, wealth quintile, and women’s education) on egg consumption. The natural effect model was specified as a binomial logistic regression model, regressing the two imputed counterfactual outcomes for each woman on egg consumption, adjusting for religion, wealth quintile, and women’s education. Mediator-outcome confounders were pre-specified based on theoretical knowledge and included in the model if they were significantly correlated with egg consumption and poultry ownership at the 5% significance level. Missing data on women’s wealth quintile was imputed using the cluster average wealth quintile, rounded to the nearest integer. We did not find evidence of an interaction between the exposure and mediator and so did not include any interaction terms in the models. We used the same model structures to test for mediation at each of the three time periods.

References:

Steen, J., Loeys, T., Moerkerke, B., & Vansteelandt, S. (2017). medflex: An R Package for Flexible Mediation Analysis using Natural Effect Models. Journal of Statistical Software, 76(11). https://doi.org/10.18637/jss.v076.i11


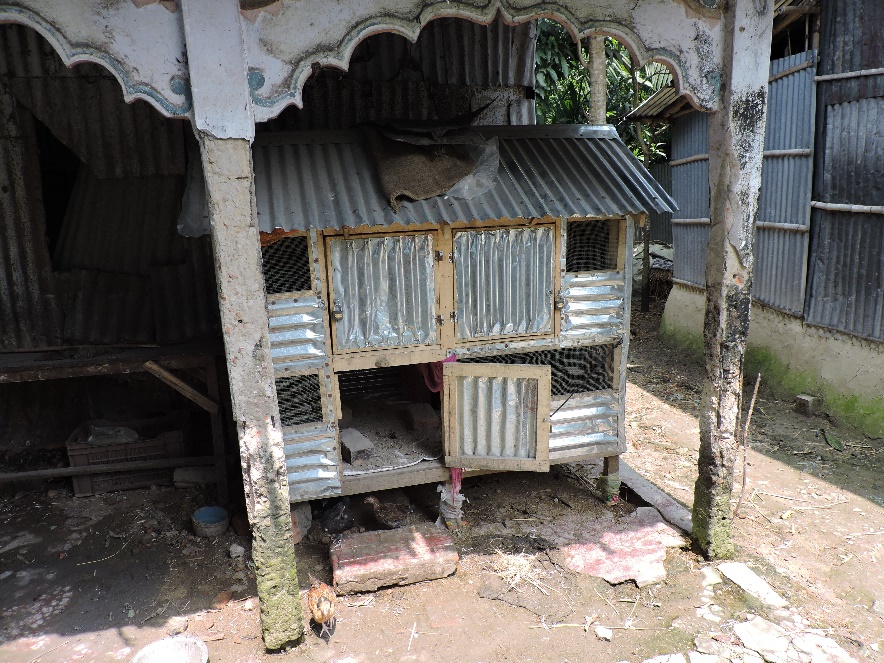


Supplemental Figure 1. Picture of an “improved” poultry shed promoted by the Homestead Food Production intervention. “Improved” poultry sheds had separate rooms for adult chickens and chicks, were elevated off the ground, well-ventilated with a window, easy to clean, secure, and were made of tin, wood, or bamboo. Photo credit: FAARM team. *Abbreviations:* FAARM, Food and Agricultural Approaches to Reducing Malnutrition.

***
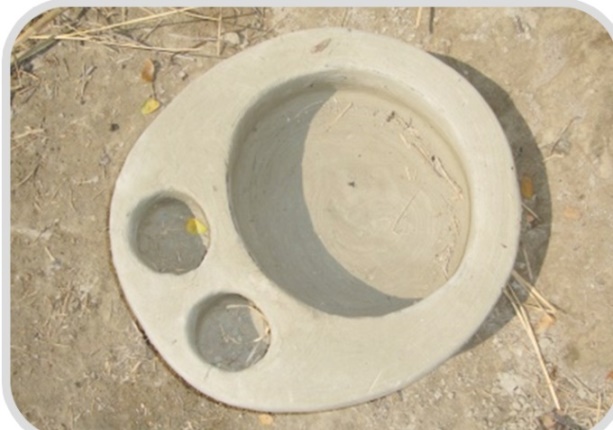
***

Supplemental Figure 2. Hatching pot (hazal). Hatching pots are made for poultry to incubate their eggs. The two smaller holes provide hens with food and water. Photo credit: FAARM team. *Abbreviations:* FAARM, Food and Agricultural Approaches to Reducing Malnutrition.

**
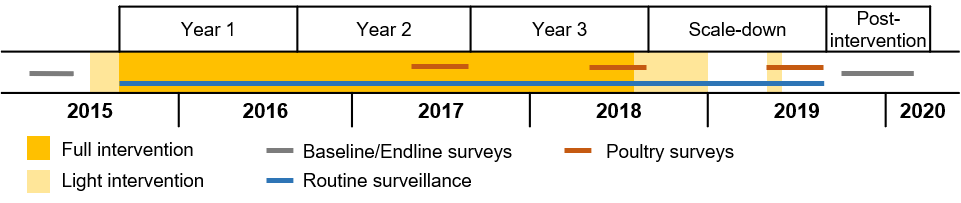
**

Supplemental Figure 3. Timeline of the FAARM trial and surveys used in the analyses. The baseline survey was conducted from March to May 2015. The first year of the intervention was from Sep. 2015 to Aug. 2016, the second year from Sep. 2016 to Aug. 2017, and third year from Sep. 2017 to Aug. 2018. The scale down of the intervention was from Sep. 2018 to Sep. 2019 during which field activities were phased out and stopped by Dec. 2018 and only a nutrition counseling refresher training was provided in May 2019. Routine assessments were conducted as part of a surveillance system from Sep. 2015 to Sep. 2019, including poultry surveys in 2017, 2018 and 2019. The post-intervention endline survey was conducted from Oct. 2019 to Feb. 2020. *Abbreviations:* FAARM, Food and Agricultural Approaches to Reducing Malnutrition.

# Supplemental Table 1. Number of observations and clusters included per round for women’s and children’s egg consumption.

| Survey round | Women | | | | Children^a^ | | | |
| --- | --- | --- | --- | --- | --- | --- | --- | --- |
|  | Control | | Intervention | | Control | | Intervention | |
|  | Obs. | Clusters | Obs. | Clusters | Obs. | Clusters | Obs. | Clusters |
| Baseline | 1,309 | 48 | 1,290 | 48 | 610 | 48 | 593 | 48 |
| 1 | 389 | 48 | 410 | 48 | 410 | 48 | 425 | 48 |
| 2 | 402 | 48 | 396 | 48 | 414 | 48 | 394 | 48 |
| 3 | 384 | 48 | 396 | 48 | 403 | 48 | 384 | 48 |
| 4 | 388 | 48 | 398 | 48 | 396 | 48 | 427 | 48 |
| 5 | 371 | 48 | 371 | 48 | 370 | 48 | 352 | 48 |
| 6 | 371 | 48 | 376 | 48 | 373 | 48 | 358 | 48 |
| 7 | 374 | 48 | 376 | 47 | 389 | 48 | 369 | 48 |
| 8 | 389 | 48 | 379 | 48 | 361 | 48 | 352 | 48 |
| 9 | 404 | 48 | 391 | 48 | 370 | 48 | 342 | 48 |
| 10 | 380 | 48 | 400 | 48 | 332 | 47 | 338 | 48 |
| 11 | 376 | 48 | 371 | 48 | 338 | 48 | 319 | 47 |
| 12 | 374 | 48 | 384 | 48 | 338 | 48 | 332 | 48 |
| 13 | 369 | 48 | 389 | 48 | 311 | 47 | 320 | 48 |
| 14 | 397 | 48 | 390 | 48 | 328 | 48 | 316 | 48 |
| 15 | 386 | 48 | 396 | 48 | 298 | 48 | 303 | 48 |
| 16 | 369 | 48 | 384 | 48 | 300 | 48 | 314 | 47 |
| 17 | 368 | 48 | 371 | 48 | 285 | 47 | 286 | 48 |
| 18 | 371 | 48 | 384 | 48 | 282 | 48 | 293 | 48 |
| 19 | 364 | 48 | 390 | 48 | 271 | 48 | 302 | 46 |
| 20 | 374 | 48 | 368 | 48 | 256 | 47 | 277 | 47 |
| 21 | 369 | 48 | 377 | 48 | 264 | 48 | 274 | 48 |
| 22 | 362 | 48 | 374 | 48 | 253 | 48 | 258 | 48 |
| 23 | 363 | 48 | 349 | 48 | 228 | 44 | 265 | 48 |
| 24 | 381 | 48 | 398 | 48 | 279 | 46 | 264 | 48 |
| Endline | 1,282 | 48 | 1,274 | 48 | 257 | 46 | 275 | 48 |
| Total | 11,666 |  | 11,782 |  | 8,716 |  | 8,732 |  |

^a^Excludes observations in which child was less than 6 months old at the time of the survey.

# Supplemental Table 2. Poultry shed ownership and characteristics of FAARM intervention and control households at endline (Oct. 2019 - Feb. 2020).

| Characteristic | Control | | | Intervention | |
| --- | --- | --- | --- | --- | --- |
|  | n | % | n | | % |
|  | N=1213 |  | N=1227 | |  |
| Owns a poultry shed | 164 | 13.5 | 666 | | 54.3 |
|  |  |  |  | |  |
| *Poultry shed characteristics* | N=154 |  | N=654 | |  |
| Traditional/unimproved shed | 46 | 29.9 | 102 | | 15.6 |
| Separate rooms for adult chickens and chicks | 10 | 6.5 | 491 | | 75.1 |
| Off the ground | 116 | 75.3 | 221 | | 33.8 |
| Well-ventilated (window) | 69 | 44.8 | 490 | | 74.9 |
| Easy to clean / mat | 82 | 53.2 | 489 | | 74.8 |
| Secure (has a lock) | 55 | 35.7 | 239 | | 36.5 |
| With hatching pot (*hazal*) | 8 | 5.2 | 131 | | 20.0 |
| With water feeder | 12 | 7.8 | 176 | | 26.9 |
| With pest prevention (neem, ash, etc.) | 1 | 0.6 | 72 | | 11.0 |
| Made of tin, wood, or bamboo | 51 | 33.1 | 522 | | 79.8 |

# Supplemental Table 3. Effect of a Homestead Food Production intervention on egg consumption among women and their children aged 6-36 months, adjusted for household wealth.

| Time period | Women | | Children | |
| --- | --- | --- | --- | --- |
|  | OR (95% CI) | P-value | OR (95% CI) | P-value |
| Intervention Y1, Sep 15 - Feb 16 | 1.22 (0.87, 1.72) | 0.24 | 1.38 (0.86, 2.22) | 0.18 |
| Intervention Y1, Mar 16 - Aug 16 | 1.16 (0.81, 1.65) | 0.42 | 1.41 (0.79, 2.51) | 0.25 |
| Intervention Y2, Sep 16 - Feb 17 | 1.80 (1.23, 2.62) | 0.002 | 0.99 (0.52, 1.90) | 0.99 |
| Intervention Y2, Mar 17 - Aug 17 | 1.62 (1.17, 2.24) | 0.004 | 1.87 (1.26, 2.77) | 0.002 |
| Intervention Y3, Sep 17 - Feb 18 | 2.08 (1.49, 2.88) | <0.001 | 2.81 (1.77, 4.47) | <0.001 |
| Intervention Y3, Mar 18 - Aug 18 | 2.22 (1.64, 3.03) | <0.001 | 2.68 (1.75, 4.10) | <0.001 |
| Scale-down, Sep 18 - Feb 19 | 1.75 (1.26, 2.42) | 0.001 | 2.17 (1.32, 3.58) | 0.002 |
| Scale-down, Mar 19 - Sep 19 | 1.70 (1.23, 2.34) | 0.001 | 1.75 (1.03, 3.00) | 0.04 |
| Post-intervention, Oct 19 - Feb 20 | 1.52 (1.13, 2.05) | 0.005 | 1.70 (0.89, 3.25) | 0.11 |
| Total observations in model | 20849 |  | 16245 |  |
| Total n in model | 2670 |  | 2992 |  |
| *Notes:* Values are OR (odds ratio) and 95% CI (confidence interval) calculated using mixed-effects logistic regression models with fixed effects for intervention group, month of survey, and Ramadan, adjusting for household wealth quintile. An interaction term was used to calculate effects by half-year. Clustering is accounted for using random effects at the settlement level. Models additionally include random effects for woman/mother and child, if applicable, to account for repeat measures. Analyses are intention-to-treat. The model for children additionally includes covariates for age, square root of age, and sex and probability weights to account for less frequent sampling of children >18 months old during surveillance rounds. | | | | |

# Supplemental Table 4. Effect of the Homestead Food Production intervention on egg consumption among children aged 6-36 months across three 1.5-year time periods.

| Time period | Model 1 | | Model 2 | |
| --- | --- | --- | --- | --- |
|  | OR (95% CI) | P-value | OR (95% CI) | P-value |
| Early intervention (Sep. 2015 - Feb. 2017) | 1.34 (0.84, 2.15) | 0.22 | 1.25 (0.80, 1.95) | 0.34 |
| Peak intervention (Mar. 2017 - Aug. 2018) | 2.60 (1.92, 3.52) | <0.001 | 2.40 (1.82, 3.17) | <0.001 |
| Scale-down/Post-intervention^a^ (Sep. 2018 - Feb. 2020) | 2.05 (1.38, 3.06) | <0.001 | 1.91 (1.30, 2.80) | 0.001 |
| Observations | 16245 |  | 16245 |  |
| n | 2992 |  | 2992 |  |
| *Notes:* Odds ratios (OR) and 95% confidence intervals (CI) were calculated using mixed-effects logistic regression models. Model 1 is adjusted for month of survey, Ramadan, child age, square root of age, and sex as well as random effects for settlement, for mother to account for similarities among siblings, and for child to account for repeat observations. Model 2 is additionally adjusted for household wealth quintile. Probability weights were used to account for less frequent sampling of children older than 18 months during surveillance rounds. An interaction term was used to calculate effects by 1.5-year periods.  ^a^The post-intervention endline survey conducted from Oct. 2019 to Feb. 2020 included only children 6-24 months old. | | | | |

# Supplemental Table 5. Associations of women’s poultry ownership with women’s and children's egg consumption at three time points during the FAARM trial.

|  | Women's egg consumption | | | | Children's egg consumption | | | |
| --- | --- | --- | --- | --- | --- | --- | --- | --- |
|  | Model 1 |  | Model 2 |  | Model 1^a^ |  | Model 2^a^ |  |
|  | OR (95% CI) | P-value | OR (95% CI) | P-value | OR (95% CI) | P-value | OR (95% CI) | P-value |
| Own ≥3 poultry (Ref: <3 poultry) |  |  |  |  |  |  |  |  |
| Intervention Year 3 | 1.28 (0.97, 1.68) | 0.08 | 1.18 (0.90, 1.55) | 0.24 | 1.35 (0.65, 2.83) | 0.42 | 1.30 (0.63, 2.73) | 0.47 |
| Scale-down | 1.27 (0.97, 1.66) | 0.08 | 1.14 (0.87, 1.49) | 0.35 | 1.73 (0.93, 3.21) | 0.08 | 1.49 (0.78, 2.86) | 0.23 |
| Post-intervention^b^ | 1.70 (1.32, 2.20) | <0.001 | 1.58 (1.22, 2.04) | <0.001 | 1.28 (0.52, 3.16) | 0.59 | 1.03 (0.40, 2.63) | 0.95 |
| Intervention group (Ref: Control) |  |  | 1.67 (1.33, 2.11) | <0.001 |  |  | 2.69 (1.76, 4.10) | <0.001 |
| Hindu religion (Ref: Muslim) |  |  | 0.59 (0.47, 0.74) | <0.001 |  |  | 0.38 (0.24, 0.61) | <0.001 |
| Household wealth quintile (Ref: Lowest) |  |  |  |  |  |  |  |  |
| Low |  |  | 1.03 (0.80, 1.33) | 0.83 |  |  | 1.29 (0.78, 2.13) | 0.33 |
| Middle |  |  | 1.15 (0.88, 1.49) | 0.30 |  |  | 1.48 (0.82, 2.68) | 0.20 |
| High |  |  | 1.24 (0.95, 1.63) | 0.12 |  |  | 2.85 (1.57, 5.19) | 0.001 |
| Highest |  |  | 1.74 (1.31, 2.31) | <0.001 |  |  | 3.47 (1.97, 6.11) | <0.001 |
| Women’s education (Ref: None) |  |  |  |  |  |  |  |  |
| Partial primary |  |  | 1.11 (0.83, 1.48) | 0.49 |  |  | 1.75 (0.88, 3.50) | 0.11 |
| Complete primary |  |  | 1.11 (0.83, 1.49) | 0.48 |  |  | 2.76 (1.43, 5.33) | 0.003 |
| Any secondary education |  |  | 1.54 (1.17, 2.04) | 0.002 |  |  | 5.53 (3.07, 9.95) | <0.001 |
| Child age, days |  |  |  |  |  |  | 0.99 (0.99, 1.00) | <0.001 |
| Square root child age |  |  |  |  |  |  | 1.43 (1.25, 1.64) | <0.001 |
| Child sex (Ref: Male) |  |  |  |  |  |  | 0.85 (0.62, 1.17) | 0.33 |
| Total observations in model | 6818 |  | 6818 |  | 3970 |  | 3970 |  |
| Total n in model | 2468 |  | 2468 |  | 1839 |  | 1839 |  |
| *Notes:* Values are OR (odds ratio) and 95% CI (confidence interval) calculated using mixed-effects logistic regression models. An interaction term was used to calculate effects by time period. Egg consumption is measured in the six months following the poultry surveillance survey. Model 1 provides unadjusted estimates and model 2 provides estimates adjusted for the covariates listed. Models include random effects for settlement, woman/mother, and child, if applicable. ^a^The models for children additionally include probability weights to account for less frequent sampling of children aged >18 months during surveillance rounds. ^b^The post-intervention survey includes only children aged 6-24 months. Abbreviations: FAARM, Food and Agricultural Approaches to Reducing Malnutrition | | | | | | | | |
